# Integrated Electrokinetics-Adsorption Remediation of Saline-Sodic Soils: Effects of Voltage Gradient and Contaminant Concentration on Soil Electrical Conductivity

**DOI:** 10.1155/2013/618495

**Published:** 2013-12-29

**Authors:** Mohammed Hussain Essa, Nuhu Dalhat Mu'azu, Salihu Lukman, Alaadin Bukhari

**Affiliations:** ^1^Department of Civil and Environmental Engineering, King Fahd University of Petroleum and Minerals, Dhahran 31261, Saudi Arabia; ^2^Department of Environmental Engineering, University of Dammam, Dammam 31451, Saudi Arabia

## Abstract

In this study, an integrated in situ remediation technique which couples electrokinetics with adsorption, using locally produced granular activated carbon from date palm pits in the treatment zones that are installed directly to bracket the contaminated soils at bench-scale, is investigated. Natural saline-sodic clay soil, spiked with contaminant mixture (kerosene, phenol, Cr, Cd, Cu, Zn, Pb, and Hg), was used in this study to investigate the effects of voltage gradient, initial contaminant concentration, and polarity reversal rate on the soil electrical conductivity. Box-Behnken Design (BBD) was used for the experimental design and response surface methodology (RSM) was employed to model, optimize, and interpret the results obtained using Design-Expert version 8 platform. The total number of experiments conducted was 15 with voltage gradient, polarity reversal rate, and initial contaminant concentration as variables. The main target response discussed in this paper is the soil electrical conductivity due to its importance in electrokinetic remediation process. Responses obtained were fitted to quadratic models whose *R* ^2^ ranges from 84.66% to 99.19% with insignificant lack of fit in each case. Among the investigated factors, voltage gradient and initial contaminant concentration were found to be the most significant influential factors.

## 1. Introduction

In situ remediation technologies for contaminated soils are faced with significant technical challenges when the contaminated soil is of low permeability [1] and possesses high electrical conductivity and exchangeable sodium percentage. Popular traditional technologies are rendered ineffective due to the difficulty encountered in accessing the contaminants as well as when employed in settings where the soil contains mixed contaminants such as petroleum hydrocarbons, heavy metals, and polar organics [2]. Though electrokinetic methods have proven to be more effective than most traditional techniques used in remediating low permeability soils and groundwater contaminated with mixed contaminants, there are still challenges—the application of optimal voltage gradient and the effective remediation of saline-sodic soil. Saline-sodic soils and groundwater aquifer formations (usually found in arid and semiarid regions) possess high electrical conductivity (4 dS/m) which prevents the application of appropriate voltage gradient in an electrokinetic study owing to current limitations [3]. In addition, these soils are associated with high pH > 8.2 and dominated by 2 : 1 type clay minerals and exchangeable sodium percentage at levels greater than 15 [4–6]. These extreme soil characteristics pose great difficulty in having such soils remediated from mixed contaminants using electrokinetics-based techniques. The usual voltage gradient of 1 V/cm for bench-scale studies when applied to such soils could lead to high electric current flow. This in turn could lead to excessive soil heating, reduction in the soil moisture content, high energy and process fluid consumption, high electroosmotic flow rate, and, in some cases, higher percentage removal of contaminants [7]. Sparks [6] posited that electrical conductivity (EC) is the best index for the assessment of soil salinity. Unfortunately, most works on electrokinetic remediation failed to at least report the soil electrical conductivity, let alone monitor its variation. Electrical conductivity greatly influences electrokinetic remediation, because it determines the amount of current flowing through the soil. EC is simultaneously influenced by many soil properties, namely, water content, soluble salts, grain size, humus, temperature, texture, and cation exchange capacity (CEC) [8].

Empirical modeling using response surface methodology (RSM) offers great and numerous advantages which include large amounts of information from a small number of experiments, evaluation of simultaneous interaction effects of the independent parameters on the responses, and simultaneous optimization of multiple factors and responses for obtaining optimal conditions [9, 10]. The key success of RSM is uncovering interactions of factors which cannot be achieved using the traditional one-factor-at-a-time (OFAT) optimization approach [11]. Fundamental understanding of the physics and chemistry which governs the process is essential in determining the influential factors to be investigated and their levels or ranges are necessary for successful implementation of RSM for any process modeling and optimization. Basically, there exist four different experimental designs for RSM implementation as follows: three-level factorial design (3FD), Box-Behnken design (BBD), central composite design (CCD), and Doehlert design (DD). Bezerra et al. [9] have reviewed each of these design methods. The Box-Behnken Design is obtained by combining two-level factorial designs with incomplete block designs followed by adding a specified number of replicated center points. BBD is preferred when investigating three factors using RSM, because it will give enough information for analyzing factor-response interactions from the least experimental runs when compared to 3FD and CCD.

In this study, an integrated in situ remediation technique which couples electrokinetics with adsorption, using locally produced granular activated carbon (GAC) from date palm pits in the treatment zones that are installed directly to bracket the contaminated soils at bench-scale, is investigated. Natural saline-sodic clay soil sampled from Al-Hasa Oasis, Saudi Arabia spiked with contaminant mixture (kerosene, phenol, Cr, Cd, Cu, Zn, Pb, and Hg), was used in this study to investigate the effects of voltage gradient, initial contaminant concentration, and polarity reversal rate on the soil electrical conductivity using RSM. Optimal conditions (factor levels) required to minimize the soil electrical conductivity were also obtained using numerical optimization.

## 2. Materials and Methods

### 2.1. Soil Characterization

Clay used in this study is a local Saudi Arabian clay from Al-Hasa Oasis. The clay pH (8.3), moisture content (3.91%), soil organic matter (2.59%), electrical conductivity (15.24 dS/m), surface area (9.07 m^2^/g), and elemental analysis using scanning electron microscopy (SEM) and X-ray diffraction (XRD) methods were determined according to the protocol outlined in the American Society of Testing and Materials (ASTM) standards and reported elsewhere [12]. The GAC used in the present study was produced locally from date palm pits as described elsewhere [13]. 1 N HNO_3_ and 2 N NaOH prepared from analytical grade reagents [purity > 99%, Sigma-Adrich] were used as catholyte and anolyte, respectively.

### 2.2. Reactor and Experimental Design

**Figure 1:**
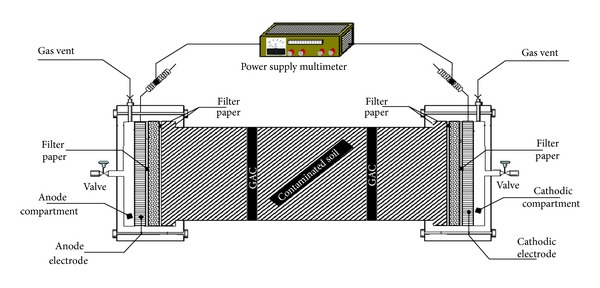
Complete experimental setup.

The plexiglass reactor total volume was about 2268 cm^3^, made of seven chambers. The overall reactor dimensions are as follows: 24 cm (long) × 10 cm (wide) × 12 cm (deep). Approximately 1 kg of local KSA soil was artificially spiked with kerosene, heavy metals (Cu, Cr, Cd, Pb, Zn, and Hg), and phenol at predetermined concentrations (see Figure 1).

Thorough mixing was done using mechanical mixer (Gilson Company Inc.) so as to achieve a homogeneous distribution of the contaminants around the soil matrix. Details of the experimental procedures are described elsewhere [7]. Box-Behnken Design (BBD) was used for the experimental design and response surface methodology (RSM) was employed to model, optimize, and interpret the results obtained using Design-Expert version 8 (Stat-Ease, Inc.) platform [11]. The total number of experiments conducted was 15 (including 3 central points) with polarity reversal rate, voltage gradient, and initial contaminant concentration (designated as *A*, *B*, and *C*, resp.) as the investigated factors which were varied according to Table 1. The main target response discussed in this paper is the soil electrical conductivity which was measured on a weekly basis for the 21-day duration of each experiment according to the method described by Sparks [6] using Accumet XL30 conductivity meter (Fisher Scientific). Other responses such as percent contaminant removal, electroosmotic flow, electric current, soil pH, moisture, organic carbon, and processing fluids refill or replacement rate were also measured.

## 3. Results and Discussions

### 3.1. Mathematical Modeling Using Response Surface Methodology (RSM)

Table 2 presents the different combinations of the factors (independent variables) based on BBD that will enable the generation of 3D response surface and contour plots. These plots depict the relative influence of the factors on electrical conductivity for proper visualization and evaluation. The weekly results of the electrical conductivity measurements are also contained in the table.

The data in Table 1 were fitted to quadratic, reduced cubic and reduced quadratic models for the 1st (1), 2nd (2), and 3rd (3) weeks EC measurement, respectively. These equations (in terms of coded factors) contain only effects or terms that are significant based on 5% significant level (from analysis of variance). All insignificant terms have been dropped, except where hierarchy is violated. Good model prediction abilities may be inferred using coefficient of determination, *R* ^2^, and lack of fit tests [11]. Equation (2) has the best *R* ^2^, followed by (1) and (3). Lack of fit for all the models is insignificant. Therefore,

(1) Y1=42.2−7.31A+32.43B−14.76C+9.25AB +13.16AC−27.76BC+9.96A2+5.4B2+13.6C2

(*R* ^2^ = 0.9766, quadratic model),

(2) Y2=70.64+30.58A+34.23B+0.82C+38.06AB −23.6AC−8.93BC+28.71A2−6.89B2 −46.12A2B−41.48A2C−61.97AB2

(*R* ^2^ = 0.9919, reduced cubic model),

(3) $$\begin{array}{c} Y_{3}=154.02+18.26A+41.94B-19.416C-64.87C^{2} \end{array}$$

(*R* ^2^ = 0.8466, reduced quadratic model), where *Y*_1_ is electrical conductivity (dS/m), 1st week; *Y*_2_ is electrical conductivity (dS/m), 2nd week; *Y*_3_ is electrical conductivity (dS/m), 3rd week; *A* is polarity reversal, hr; *B* is voltage gradient, V/cm; *C* is concentration, mg/kg.

**Figure 2:**
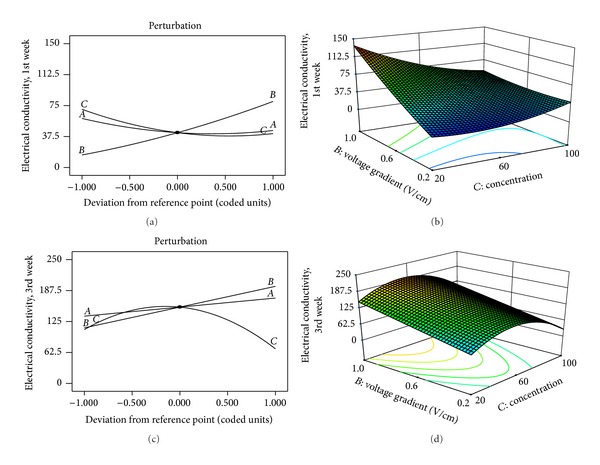
Perturbation plots showing the relative significance of factors on electrical conductivity (left). 3D response surface and contour plots showing the relative influence of factors on electrical conductivity (right): (a) and (b) 1st week; (c) and (d) 3rd week.

The perturbation plot is useful in comparing the relative effects of all factors on the response using a particular point in the design space (midpoint), with steeper slope or curvature in a factor signifying higher sensitivity of response to that factor. From Figures 2(a) and 2(c), voltage gradient (*B*) and concentration (*C*) have the steepest slope and curvature and hence have the greatest impact on the response. These influential factors became the best choice for the axes of the 3D response surface and contour plots as presented in Figures 2(b) and 2(d). Initial contaminant concentration affects the EC directly by increasing the available soluble ions in the soil, thereby enhancing its electrical conduction. The dependence of EC on factors such as soluble salts, water content, and temperature that directly affects the electrical current makes voltage gradient influential on the EC as shown in Figures 2(a) and 2(c).

### 3.2. Numerical Optimization Using Desirability Function

Numerical optimization module in Design-Expert software searches for a combination of factor levels that simultaneously satisfy the goals placed on the operating parameters (factors and responses). These goals are then combined into an overall desirability function which ranges from 0 (i.e., outside of the limits) to 1 (i.e., at the goal). The program utilizes numerical optimization algorithms to find a point(s) that maximizes the desirability function not to clinch a desirability value of 1 but to find a good set of conditions that will meet all the set goals for each factor and response. Simply put, desirability is one of the mathematical methods for computing optimum values [14]. Overall desirability of 0.64 was obtained after minimizing the weekly EC (Table 3).

The optimization results presented in Table 3 suggest a steady increase in the EC with time. Similar trend is also discernible from most of the experimental runs in Table 2. The saline-sodic nature of the soil necessitates the use of processing fluids to continuously neutralize the rapidly generated H^+^ and OH^−^ ions at the anode and cathode, respectively. These fluids were monitored every 8 hours and replaced as they deteriorated. HNO_3_ and NaOH are strong acid and base, respectively, and they dissociate completely according to the following reactions:

(4) $$\begin{array}{c} \text{HN}{\text{O}}_{3}\;\left(\text{l}\right)\to {\text{H}}^{+}\;\left(\text{aq}\right)+{{\text{NO}}_{3}}^{-}\;\left(\text{aq}\right),\\ \text{NaOH}\;\left(\text{aq}\right)\to \text{N}{\text{a}}^{+}\;\left(\text{aq}\right)+\text{O}{\text{H}}^{-}\;\left(\text{aq}\right). \end{array}$$

As a result of the electrochemical decomposition of water, OH^−^ and H^+^ ions are produced at the cathode and anode, respectively, as shown in

(5) $$\begin{array}{c} 4{\text{H}}_{2}\text{O}\;\left(\text{l}\right)+4{\text{e}}^{-}\to {\text{H}}_{2}\;\left(\text{g}\right)+4\text{O}{\text{H}}^{-}\;\left(\text{aq}\right), \end{array}$$

(6) $$\begin{array}{c} 2{\text{H}}_{2}\text{O}\;\left(\text{l}\right)\to {\text{O}}_{2}\;\left(\text{g}\right)+4{\text{H}}^{+}\;\left(\text{aq}\right)+4{\text{e}}^{-}. \end{array}$$

The electrochemically generated H^+^ and OH^−^ ions due to water electrolysis at the anode and cathode, respectively, are neutralized to form water molecules (7) as a result of the OH^−^ and H^+^ ions produced from the dissociation of the catholyte and anolyte, respectively, as shown in (4). So,

(7) $$\begin{array}{c} {\text{H}}^{+}\;\left(\text{aq}\right)+\text{O}{\text{H}}^{-}\;\left(\text{aq}\right)\to {\text{H}}_{2}\text{O}\;\left(\text{l}\right). \end{array}$$

The oxygen and hydrogen gases generated may be vented out, while some amount may go into the soil and alter the redox chemistry. Na^+^ and NO_3_^−^ ions migrate into the soil to the opposite electrodes, thereby increasing the electrical conductivity as the treatment process progresses. A sustained and variable electroosmotic flow was observed due to the migration of the Na^+^ ions which could enhance the migration of the double layer complexes toward the cathode, while nitrate ions could be involved in complex formation with the cations.

Since the processing fluids are finite in volume and the electrochemical decomposition of water at the electrodes is continuous, then a time will be reached when all the ions in the processing fluids have been exhausted. Consequently, rise and fall in catholyte pH and anolyte pH, respectively, are expected before the replacement of the processing fluids. At this juncture, OH^−^ ions generated at the cathode according to (5) migrate into soil toward the anode. In this migration process, soil pH rises and metal hydroxides are formed which could precipitate and reduce the electrical conductivity and increase current consumption near the cathode. At the same time, soluble hydroxo complexes are formed with the cations due to complexing property of the hydroxyl ions. On the other hand, hydrogen ions generated at the anode (6) migrate toward the cathode. This process may lead to soil protonation or desorption of indigenous and spiked heavy metals, hence increasing the electrical conductivity. Given the presence of carbonates in the soil minerals, the developing acid front may be buffered by the carbonate minerals, thereby hindering any fall in the soil pH. From the forgoing discussion, it is clear that there will be an increase in the soil electrical conductivity as the integrated electrokinetics-adsorption remediation progresses. In addition, the electroosmotic flow will undoubtedly vary spatially and temporally as it also depends on the soil zeta potential, pore fluid viscosity, and permittivity.

## 4. Conclusions 

The results presented have highlighted and elucidated the extent of the influence of voltage gradient and concentration on electrical conductivity using mathematical models and numerical optimization. The empirical models developed in this study may be used for preliminary estimation of soil electrical conductivity; however, actual soil under study should be tested for verification. The models are limited to the derived results obtained for saline-sodic soil. Hence, generalization and use of these models for different soil types and operating conditions may lead to incorrect estimation of the electrical conductivity. From the perturbation plot, the following sequence of relative influence of the operating parameters on EC can be inferred: concentration > voltage gradient > polarity reversal. Hence lower voltage gradient may lead to lower electrical current, temperature, energy consumption, and consequently lower electrical conductivity. On the other hand, high electrical conductivity was found to allow high electrical current through the soil and eventual rise in the cost of operating the remediation process.

## Figures and Tables

**Table 1:** Information on the independent variables.

Variable	Designation	Units	Coded variable levels		
			−1	0	+1
Polarity reversal	*A *	Hours	0	24	48
Voltage gradient	*B *	V/cm	0.2	0.6	1
Concentration	*C *	mg/kg	20	60	100

**Table 2:** Weekly results of electrical conductivity based on Box-Behnken Design.

Run order	Polarity reversal, *A *	Voltage gradient, *B *	Concentration, *C *	Electrical conductivity dS/m		
				1st week	2nd week	3rd week
1	24	0.6	60	42.2	74.5	150.1
2	0	0.2	60	48.72	176.7	117.3
3	0	0.6	20	102.6	82.93	105.5
4	48	0.6	20	64.4	191.3	147.7
5	24	0.6	60	37.98	67.05	135.09
6	48	0.2	60	12.85	37.8	94.75
7	24	0.6	60	46.42	81.95	165.11
8	48	1	60	84.9	90.12	221.9
9	24	1	20	138.8	103.2	129.1
10	24	0.2	20	7.14	16.88	51.98
11	24	0.2	100	39.11	36.39	37.3
12	0	1	60	83.78	76.8	193.9
13	0	0.6	100	40.8	48.82	25.64
14	48	0.6	100	55.23	62.77	124.1
15	24	1	100	59.74	86.98	91.93

**Table 3:** Results of numerical optimization of factors and responses using desirability.

Item	Value
Polarity reversal, hours	21.61
Voltage gradient, V/cm	0.51
Concentration, mg/kg	76.02
1st week electrical conductivity, dS/m	34.59
2nd week electrical conductivity, dS/m	63.20
3rd week electrical conductivity, dS/m	124.88

Desirability	0.64
